# Cardiomiopatia Diabética: Desvendando Mecanismos Fisiopatológicos por Meio de RNAs não Codificantes

**DOI:** 10.36660/abc.20250395

**Published:** 2025-08-06

**Authors:** Elida Paula Benquique Ojopi, Éder Anderson Rodrigues, Gustavo Augusto Ferreira Mota, Marina Politi Okoshi, Luana Urbano Pagan

**Affiliations:** 1 Hospital das Clínicas da Faculdade de Medicina de Botucatu Botucatu SP Brasil Hospital das Clínicas da Faculdade de Medicina de Botucatu, Botucatu, SP – Brasil; 2 Universidade Estadual Paulista Júlio de Mesquita Filho Faculdade de Medicina Departamento de Clínica Médica Botucatu SP Brasil Universidade Estadual Paulista Júlio de Mesquita Filho, Faculdade de Medicina – Departamento de Clínica Médica, Botucatu, SP – Brasil

**Keywords:** Cardiomiopatias Diabéticas, MicroRNAs, RNA Longo não Codificante

A cardiomiopatia diabética (CMD) é uma complicação grave do diabetes mellitus (DM) caracterizada por alterações que resultam em remodelamento cardíaco com disfunção ventricular e, eventualmente, insuficiência cardíaca, na ausência de outras condições como hipertensão arterial, valvopatias, doença arterial coronariana e cardiopatias congênitas.^1^ A CMD se desenvolve independentemente de outros fatores de risco cardiovasculares e está associada a alterações metabólicas, incluindo hiperglicemia e hiperlipidemia. Vários mecanismos contribuem para a patogênese da CMD, tais como sinalização insulínica cardíaca desregulada, disfunção mitocondrial, estresse oxidativo, inflamação e alterações no equilíbrio de cálcio.^2^ Apesar de sua crescente prevalência, os mecanismos pelos quais a CMD se desenvolve permanecem amplamente não elucidados, e um tratamento eficaz ainda é necessário.^3^

RNAs não codificantes (ncRNAs) são produtos da transcrição que não são traduzidos em proteínas, apresentando uma ampla diversidade quanto à biogênese, tamanho, forma e função. Os ncRNAs mais extensivamente estudados são os microRNAs (miRNAs) e os RNAs longos não codificantes (lncRNAs). Atualmente, é amplamente aceito que ncRNAs com mais de 200 nucleotídeos de comprimento são classificados como lncRNAs. Os miRNAs pertencem a uma classe mais ampla de ncRNAs conhecida como "pequenos RNAs", definidos por seu tamanho (20–28 nucleotídeos) e associação com proteínas da família Argonauta (Ago). Esses pequenos RNAs atuam como guias, direcionando as proteínas Ago para ácidos nucleicos-alvo, a fim de induzir o silenciamento gênico.^4^ miRNAs e lncRNAs regulam diversos processos envolvidos na CMD, incluindo estresse oxidativo, inflamação, apoptose e autofagia. Por isso, têm sido explorados como potenciais biomarcadores e alvos terapêuticos.^5 , 6^

H19 é um lncRNA identificado em 1990^7^ como um dos transcritos com *imprinting* materno. Possui 2,3 kb de comprimento, é abundantemente expresso e apresenta características de mRNA, exceto pela ausência de potencial codificador. Transcrito pela RNA polimerase II (RNAPII) e localizado no citoplasma, o H19 passa por *capping* , *splicing* e poliadenilação.

Nesta edição dos Arquivos Brasileiros de Cardiologia, Wang et al.^8^ investigaram os efeitos cardioprotetores da orientina, um flavonoide com propriedades antioxidantes,^9 , 10^ com foco no papel dos ncRNAs, especificamente no eixo H19/miR-103-3p/ALDH2/PI3K/AKT. Este estudo interessante avança ao conectar um composto natural a mecanismos moleculares finamente regulados na fisiopatologia da CMD.

A CMD foi induzida em camundongos por meio da combinação de uma dieta rica em gorduras e injeções de estreptozotocina. A pesquisa envolveu vários grupos de tratamento para avaliar os efeitos de diferentes doses de orientina (10 mg/kg, 20 mg/kg e 40 mg/kg por dia, por via intraperitoneal) ao longo de 12 semanas.^8^ Apesar dos níveis de glicose no sangue permanecerem inalterados, houve redução significativa da fibrose miocárdica e da apoptose, além de melhora da função cardíaca nos animais tratados. A orientina reduziu os níveis de marcadores bioquímicos de dano cardíaco, como LDH, CK-MB e cTnI. Os resultados sugeriram que a orientina aumentou a atividade de enzimas antioxidantes, uma vez que o pré-tratamento inibiu a produção de espécies reativas de oxigênio (ROS) induzida por alta glicose em cardiomiócitos HL-1. O estudo destacou que os níveis de lncRNA H19 e ALDH2 estavam reduzidos, enquanto miR-103-3p estava aumentado na condição diabética. A orientina reverteu essas alterações, indicando que modula o eixo de sinalização H19/miR-103-3p/ALDH2, o qual desempenha um papel crucial na fisiopatologia da CMD. A orientina também ativou a via de sinalização PI3K/AKT, essencial para a sobrevivência celular, associada à regulação positiva de ALDH2, sugerindo um mecanismo protetor contra o estresse oxidativo e a apoptose em cardiomiócitos. Especificamente, H19 aumentou a expressão de ALDH2 ao se ligar ao miR-103-3p, um inibidor de ALDH2, ativando assim a via PI3K/AKT em células HL-1 tratadas com alta glicose.

Por fim, experimentos de "resgate" (silenciamento de H19 e inibição de PI3K) demonstraram que a depleção de H19 ou a inibição de PI3K reverteram os efeitos protetores da orientina, estabelecendo uma relação causal entre esse eixo molecular e os benefícios observados. Os resultados deste estudo reforçam as propriedades cardioprotetoras da orientina, previamente sugeridas em outros contextos, como no infarto do miocárdio.^11^

Investigações futuras sobre os alvos diretos de miR-103-3p podem proporcionar uma compreensão mais aprofundada da rede regulatória envolvida na CMD. Além disso, estudos translacionais em modelos animais maiores e, futuramente, ensaios clínicos em pacientes com CMD, serão cruciais para validar o potencial terapêutico da orientina e a relevância clínica do eixo H19/miR-103-3p/ALDH2/PI3K/AKT como alvo terapêutico.
