# Supplementary material for: Cardiomiopatia Diabética: Desvendando Mecanismos Fisiopatológicos por Meio de RNAs não Codificantes
Source: Arq Bras Cardiol. 2025 Aug 6;122(7):e20250395. [Article in Portuguese] doi: 10.36660/abc.20250395 (PMC12695056; doi:10.36660/abc.20250395)
Supplement: Translation

# Translation: Diabetic Cardiomyopathy: Unraveling Pathophysiological Mechanisms via Non-coding RNAs

**DOI:** 10.36660/abc.20250395i

**Authors:** Elida Paula Benquique Ojopi, Éder Anderson Rodrigues, Gustavo Augusto Ferreira Mota, Marina Politi Okoshi, Luana Urbano Pagan

**Affiliations:** 1 Botucatu SP Brazil Hospital das Clínicas da Faculdade de Medicina de Botucatu, Botucatu, SP – Brazil; 2 Botucatu SP Brazil Universidade Estadual Paulista Júlio de Mesquita Filho, Faculdade de Medicina – Departamento de Clínica Médica, Botucatu, SP – Brazil

**Keywords:** Diabetic Cardiomyopathies, MicroRNAs, Long Noncoding RNA

Diabetic cardiomyopathy (DCM) is a serious complication of diabetes mellitus (DM) characterized by changes that result in cardiac remodeling with ventricular dysfunction and eventually heart failure in the absence of other conditions such as arterial hypertension, valvular heart disease, coronary artery disease, and congenital heart disease.^1^DCM develops independently of other cardiovascular risk factors and is associated with metabolic abnormalities, including hyperglycemia and hyperlipidemia. Multiple mechanisms contribute to DCM pathogenesis, such as impaired cardiac insulin signaling, mitochondrial dysfunction, oxidative stress, inflammation, and calcium handling defects.^2^Despite its increasing prevalence, the underlying mechanisms of DCM remain largely unelucidated, and effective treatment is still needed.^3^

Non-coding RNAs (ncRNAs) are transcriptional outputs that do not translate into proteins, exhibiting a diverse range of biogenesis, size, shape, and function. The most extensively studied ncRNAs are microRNAs (miRNAs) and long non-coding RNAs (lncRNAs). It has become a broadly accepted definition that ncRNAs over 200 nucleotides in length are classified as lncRNAs. miRNAs belong to a broader class of ncRNAs known as ‘small RNAs’, which are defined by their size (20–28 nucleotides) and association with Argonaute (Ago) family proteins. Small RNAs act as guides, directing Ago proteins to target nucleic acids to induce gene silencing.^4^miRNAs and lncRNAs regulate various processes involved in DCM, including oxidative stress, inflammation, apoptosis, and autophagy. Therefore, they have been explored as potential biomarkers and therapeutic targets.^5 , 6^

H19 is a lncRNA identified in 1990^7^as one of the maternally imprinted transcripts. H19 is 2.3 kb in length, abundantly expressed, and has characteristics of mRNA except that it lacks coding potential. Transcribed by RNA polymerase II (RNAPII) and localized in the cytoplasm, H19 undergoes capping, splicing, and polyadenylation.

In this issue of *Arquivos Brasileiros de Cardiologia* , Wang et al.^8^investigated the cardioprotective effects of orientin, a flavonoid with antioxidant properties,^9 , 10^with a focus on the role of ncRNAs, specifically the H19/miR-103-3p/ALDH2/PI3K/AKT axis. This interesting study advances by connecting a natural compound with finely regulated molecular mechanisms to DCM pathophysiology.

DCM was induced using a combination of a high-fat diet and streptozotocin injections in mice. The research involved multiple treatment groups to assess the effects of different doses of orientin (10 mg/kg, 20 mg/kg, and 40 mg/kg per day intraperitoneally) over 12 weeks.^8^Despite unchanged blood glucose levels, myocardial fibrosis and apoptosis were significantly reduced, and cardiac function improved in the treated mice. Orientin reduced the levels of biochemical markers associated with cardiac damage, such as LDH, CK-MB, and cTnI. Results suggested that orientin increased antioxidant enzyme activities as pretreatment inhibited high glucose-triggered ROS production in HL-1 cardiomyocytes. The study highlighted that lncRNA H19 and ALDH2 levels were reduced while miR-103-3p increased in diabetic conditions. Orientin reversed these changes, indicating that it modulates the H19/miR-103-3p/ALDH2 signaling axis, which plays a crucial role in the pathophysiology of DCM. Orientin also activated the PI3K/AKT signaling pathway, essential for cell survival, which was linked to ALDH2 upregulation, suggesting a protective mechanism against oxidative stress and apoptosis in cardiomyocytes. Specifically, H19 upregulated ALDH2 expression by binding to miR-103-3p, an ALDH2 inhibitor, thereby activating the PI3K/AKT pathway in high glucose-treated HL-1 cells.

Finally, "rescue" experiments (H19 knockdown and PI3K inhibition) demonstrated that H19 depletion or PI3K inhibition reversed the protective effects of orientin, establishing a causal link between this molecular axis and the observed benefits. The results of this study reinforce the cardioprotective properties of orientin previously suggested in other contexts, such as in myocardial infarction.^11^

Future investigations into the direct targets of miR-103-3p can provide a better understanding of the regulatory network involved in DCM. Furthermore, translational studies in larger animal models and, eventually, clinical trials in DCM patients would be crucial to validate the therapeutic potential of orientin and the clinical relevance of the H19/miR-103-3p/ALDH2/PI3K/AKT axis as a therapeutic target.
